# Intestinal barrier functions in hematologic and oncologic diseases

**DOI:** 10.1186/s12967-023-04091-w

**Published:** 2023-03-31

**Authors:** Elio Haroun, Prashanth Ashok Kumar, Ludovic Saba, Joseph Kassab, Krishna Ghimire, Dibyendu Dutta, Seah H. Lim

**Affiliations:** 1 Division of Hematology and Oncology, State University of New York Upstate Medical University, SUNY Upstate Medical University, 750 E Adams, Syracuse, NY 13210 USA; 2 Department of Medicine, Saint-Joseph University of Beirut, Beirut, Lebanon

**Keywords:** Intestinal barrier, Hematologic, Oncologic, Complications, Pathogenesis

## Abstract

The intestinal barrier is a complex structure that not only regulates the influx of luminal contents into the systemic circulation but is also involved in immune, microbial, and metabolic homeostasis. Evidence implicating disruption in intestinal barrier functions in the development of many systemic diseases, ranging from non-alcoholic steatohepatitis to autism, or systemic complications of intestinal disorders has increased rapidly in recent years, raising the possibility of the intestinal barrier as a potential target for therapeutic intervention to alter the course and mitigate the complications associated with these diseases. In addition to the disease process being associated with a breach in the intestinal barrier functions, patients with hematologic and oncologic diseases are particularly at high risks for the development of increased intestinal permeability, due to the frequent use of broad-spectrum antibiotics and chemoradiation. They also face a distinct challenge of being intermittently severely neutropenic due to treatment of the underlying conditions. In this review, we will discuss how hematologic and oncologic diseases are associated with disruption in the intestinal barrier and highlight the complications associated with an increase in the intestinal permeability. We will explore methods to modulate the complication. To provide a background for our discussion, we will first examine the structure and appraise the methods of evaluation of the intestinal barrier.

## Background

The gastrointestinal (GI) mucosal epithelium is one of the largest tissue systems that maintain constant interaction with the external environment. It forms an important physical and functional barrier separating the host internal milieu from potentially harmful and toxic entities. In addition to selectively controlling the traverse of luminal contents into the systemic circulation, GI mucosal epithelium also regulates adaptive immune responses. The physical barrier arisen from the GI mucosa is selectively permeable. It allows the absorption of nutrients and water but prevents the translocation of luminal bacteria and bacterial products into the host. The barrier may be disrupted due to mucosal injury, changes in cellular molecules, or dysregulated immune responses from autoimmune diseases.

Disruption of the intestinal barrier has been implicated in various disease states. They include inflammatory bowel disease [1], celiac disease [2], non-alcoholic steatohepatitis [3], diabetes mellitus [4], and autism [5]. It has been postulated that the tissue injury in these diseases is mediated by immune and non-immune responses to an uncontrolled influx of antigens and luminal bacterial products that occurs when the intestinal barrier is breached [6]. Although the pathogenetic effects of increased intestinal permeability has been widely discussed in many diseases, their roles in hematologic and oncologic diseases are less examined. There is a paucity of literature that explores how the disruption of intestinal barrier functions resulting from these conditions and/or their treatment impacts the disease phenotypes and complications experienced by the patients.

The intestinal barrier may, therefore, be a target for therapeutic approaches to alter the course and mitigate the complications associated with a disruption in its functions. In this paper, we will discuss how hematologic and oncologic diseases are associated with disruption in the intestinal barrier and explore the consequences of an increase in the intestinal permeability in these patients. We will also propose approaches to modulate this intestinal pathology. To do so, we will first provide an overview of the structure and methods of evaluation of the intestinal barrier as the background on which discussions on hematologic and oncologic diseases will be based.

## Main text

### Anatomy of the intestinal barrier

#### Intestinal barrier components

**Fig. 1:**
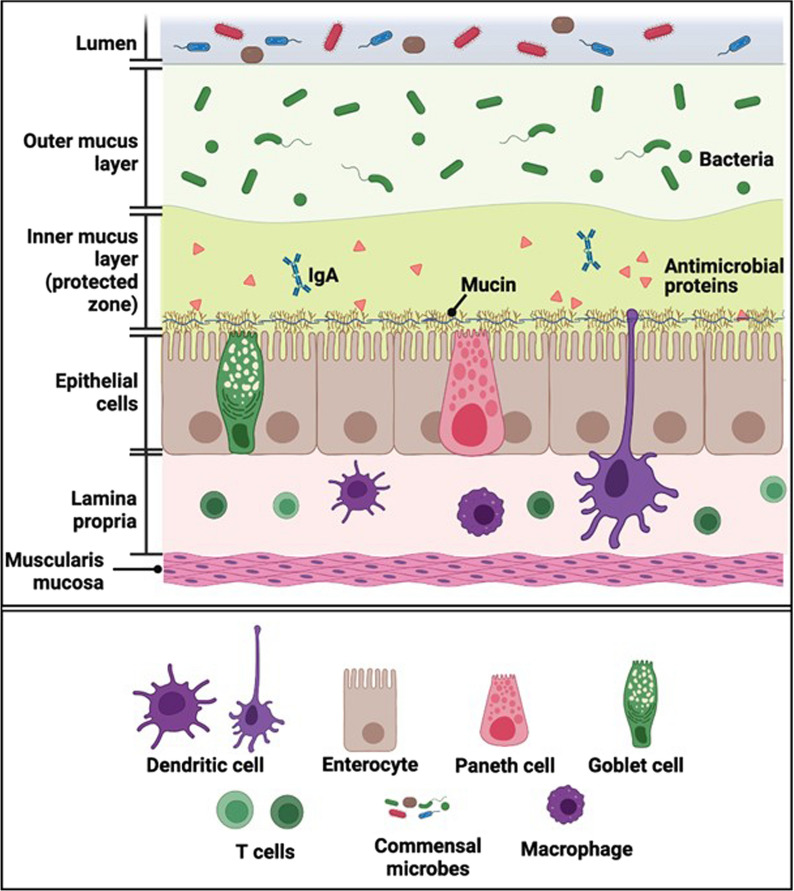
The three interactive layers of intestinal barrier. The luminal layer consists of the intestinal commensal organisms that spatially inhibit colonization of the intestine with pathogenic microbial species. The middle layer consists of mucus that prevents the contact of the mucosal epithelium with the intestinal micro-organisms. This mucus layer not only provides a physical barrier, it also contains antimicrobial proteins and IgA, both of which contribute further to protect the mucosal epithelium. The mucosal epithelium provides a selectively permeable barrier to the luminal contents

The intestinal barrier is broadly composed of three interactive layers (Fig. 1). The first layer is the luminal layer that consists of commensal microbes. These commensals inhibit the colonization of opportunistic and pathogenic microbial species. A balanced intestinal microbial community is essential for the maintenance of this layer of the barrier.

The second layer consists of unstirred water, glycocalyx and mucus. Secreted mucus mixes with antibacterial agents such as lysozyme, immunoglobulin A (IgA) and defensins produced by Paneth cells and enterocytes to confer a homeostatic environment. Enterocytes secrete transmembrane mucins that remains attached to the cell membrane and covers the apical surface for protection and sensing host-microbe interaction for innate immune response [7]. The more abundant and dynamic gel-forming mucin, mucin 2 (MUC 2), is produced by goblet cells [8]. The outer mucus layer is dynamic and has an abundance of microbes and microbial products, while the inner mucus layer remains attached to the epithelium and is more concentrated in secreted antimicrobial peptides that reduce antigen exposure to the underlying enterocytes. The mucus layer also protects the underlying epithelial layer from dehydration and mechanical stress, removes debris and microbes by flushing them away, and forms a diffusion barrier for the passage of ions, water, nutrients, and gases [9, 10]. Gut microbiota composition and mucus layer has a bidirectional interaction in which commensals promote mucus secretion and thickness, and pathogens degrade mucus.

The final component of the intestinal barrier is the epithelial layer. Enterocytes are connected by intercellular junctional complexes where tight junctions (TJs), adherens junctions and desmosomes regulate epithelial barrier function and transport. The Paneth cells in the epithelium produce defensins and other antimicrobial peptides. Underneath the epithelium, the lamina propria provides defense based on innate and acquired immunity cells such as dendritic cells, B-cells, T-cells and macrophages. Depletion of the mucus layer due to dysbiosis brings microbiota to close proximity of the epithelial cells. Pattern recognition receptors (PRRs) such as Toll-like receptors (TLRs) expressed on epithelial cells interact with microbiota-derived pathogen-associated-molecular patterns (PAMPs) such as lipopolysaccharide (LPS) to activate myeloid differentiation factor 88 (MYD88)-dependent signaling. In addition to PAMPs, damage-associated-molecular patterns (DAMPs) released from damaged intestinal epithelium interact with PRRs to facilitate macrophage- and dendritic cell-triggered inflammation. Dysbiosis can also lead to apical junction complex dysfunction. For example, excess growth of *E. coli* strain C25 increases gut permeability [11], spreading luminal content into the systemic circulation and triggering local and systemic immune response. Changes in intestinal microbiota in the form of increases in α-diversity and increase in the abundance of non-commensals or less dominant taxa correlate with an increased intestinal permeability [12].

### Intestinal permeability

**Fig. 2:**
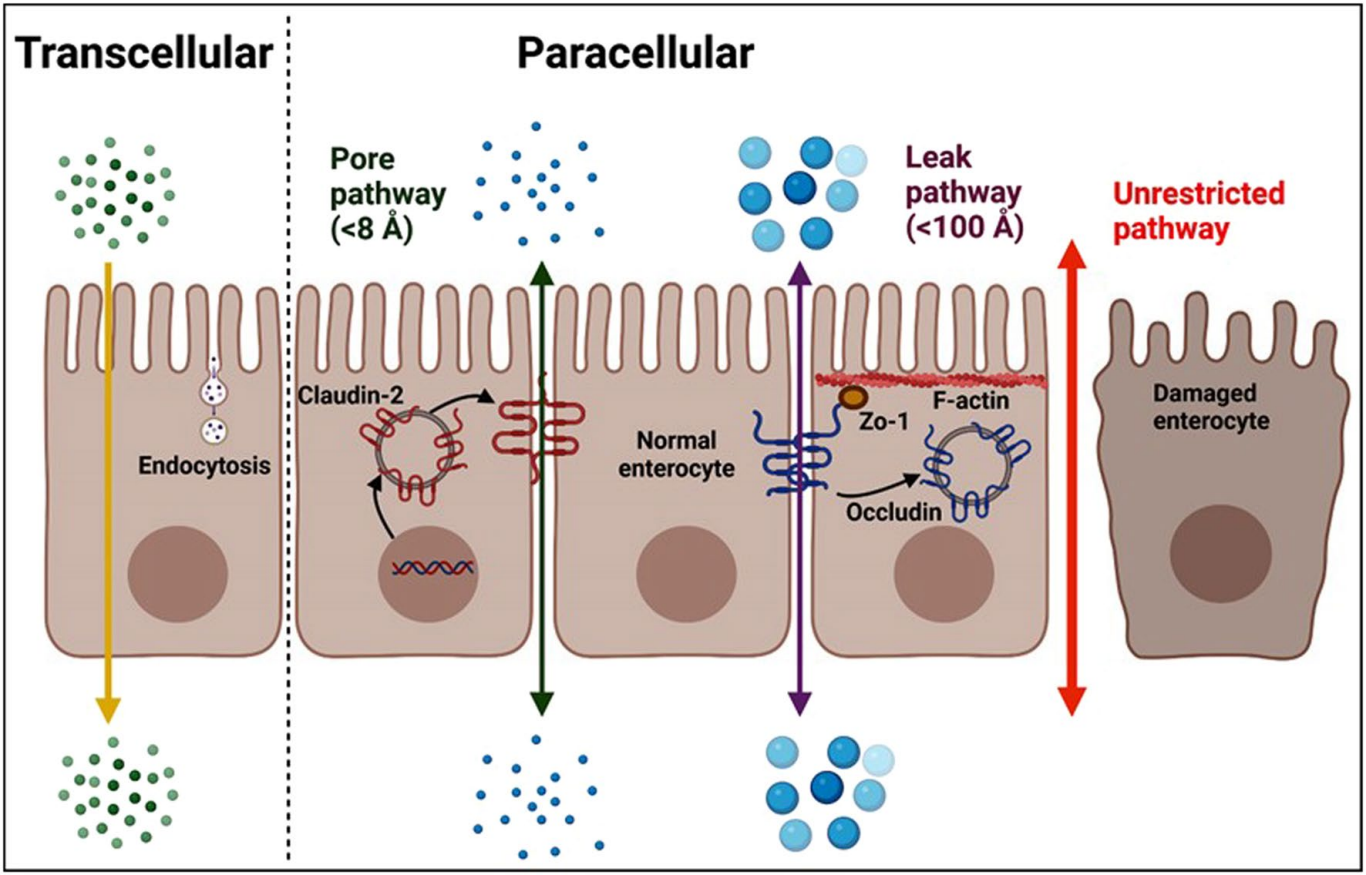
Mechanisms of intestinal barrier functions. Traverse of intraluminal content occurs across tight junctions by two different routes termed pore and the leak pathways. The pore pathway is a high-capacity, charge-selective route that regulates the passage of small molecules less than 8 Å in diameter. The leak pathway is a low-capacity, non-charge selective route traversed by molecules up to 100 Å in diameter. When epithelial damage occurs, the tight junction-independent, size-nonselective, charge-nonselective unrestricted pathway becomes the dominant route of intestinal permeability

While epithelial cells are interconnected by the apical junction complex to create a barrier, the seal is not absolute. The intestinal barrier does permit the passage of selected luminal molecules via either the paracellular pathway or the transcellular pathway (Fig. 2).

#### Paracellular pathway

The paracellular pathway provides the passage of luminal molecules between adjacent cells via TJs or intercellular spaces [13]. There are three distinct paracellular pathways: (1). The pore pathway, a high-conductance route, is charge- and size-selective (upper limit of 6 to 8 Å diameter) [13]; (2). The leak pathway, a lower conductance route, allows the movement of molecules with non-selective charge, upper size limit of ∼100 Å diameter [14]; and, (3). The unrestricted pathway, due to epithelial cell damage, is independent of TJs and allows for the pathologic flux of luminal content across the epithelium.

##### Pore pathway

Water, ions, and nutrients are transported via the pore pathway. TJ regulation of the pore pathway is dependent on various factors. Physiological Na^+^-glucose cotransport activation in intestinal epithelia enhances pore pathway permeability by activating the myosin light chain kinase (MLCK) [15]. Other modes of pore pathway regulation are governed by charge-selectivity and regulation of claudin 2 expression. Claudin 2 upregulation increases pore pathway permeability. Other members of the claudin family including 10a, 10b, 15, 16, and 17 also form either cation- or anion-selective channels [13]. Pore pathway permeability is sensitive to cytokine signaling. For example, interleukin 13 (IL-13) selectively induce claudin 2 expression to enhance pore pathway permeability [16].

##### Leak pathway

The specific site of leak pathway mediating macromolecular flux is considered to be the tricellular TJs [17]. Constitutively active MLCK expression increases leak pathway permeability [18]. Among the TJ proteins, occludin and ZO-1 are critical mediators of the leak pathway. MLCK-dependent occludin endocytosis via a caveolar process promotes leak pathway permeability [19]. Since ZO-1directly binds to F-actin [20] and the actin binding region of ZO-1 is required for barrier regulation by MLCK [21], ZO-1 downregulation or knockdown increases leak pathway permeability [22]. Among the cytokines, tumor necrosis factor (TNF) and IL-1β regulate leak pathway by promoting occludin endocytosis and MLCK activation [19, 23].

##### Unrestricted pathway

This pathway arises due to epithelial damage. This is the pathway for the translocation of luminal endotoxins, exotoxins, and intact enteric bacteria.

#### Transcellular pathway

Lipids and lipophilic substances cross the intestinal barrier via diffusion along the apical plasma membrane of enterocytes into the circulation by the transcellular pathway. In addition, large molecules, antigens and microbes can also pass through by endocytosis and transcytosis. Both endocytosis and transcytosis can be manipulated by microbes to establish their entry and translocation. M-cells in the epithelium can take up antigens and microbes by specific cell surface receptors. For example, GP2 receptor can identify specific antigens on the surface of microbes and initiate endocytosis, transcytosis, exocytosis, and engulfment by subepithelial dendritic cells [24, 25]. The goblet cell-associated antigen pathways can also aid in the internalization of endotoxins [26].

### Intestinal permeability assessment in the clinics

Table 1 summarizes the methods available, primarily on a research basis, to study intestinal permeability in human and the obstacles associated with each of the methods. By far, the method most commonly used and widely available for assessing intestinal permeability in the clinic and outside the context of research is the lactulose: mannitol (L: M) test. This is a quantitative assay to measure the ability of these two non-metabolized sugar molecules to permeate the intestinal mucosa. Mannitol, a readily absorbed monomer, is absorbed and excreted in the urine while lactulose, a dimer, is only slightly-absorbed and serves as a marker for mucosal integrity [27]. An elevated L: M ratio is an indicator of intestinal barrier dysfunction. Despite being a non-invasive and quantitative method, studies have shown significant limitations in its use [28]. Other probes used include polyethylene glycol (PEG) and radioactive chromium complexed with ethylene diamine tetracetic acid (^51^Cr-EDTA).

**Table 1:** Intestinal permeability evaluation methods

Method	Basis	Strengths	Weaknesses
Lactulose: Mannitol test	Both lactulose and mannitol are non-metabolized sugar molecules, but only mannitol is significantly absorbed following oral administration because it is smaller. The ratio of lactulose to mannitol measured in the urine provides an index of the permeability of the intestinal barrier. The higher the L: M ratio, the leakier is the intestinal barrier	Easy and inexpensive to administer and can be readily performed in the clinic without special training. Non-invasive	Normal range in healthy individuals still require to be established. Longitudinal collection of urine may be challenging in some patients, e.g. infants
Low molecular weight polyethylene glycol (PEG 400)	PEG 400 (range of molecular weight 232 to 594) is a mixture of water-soluble molecules of different sizes. It is not degraded by intestinal bacteria, and not metabolized after absorption. It is rapidly excreted in the urine. Measurements of the PEG of different molecular weight provide a characterization of the passive permeability of the mucosa		
^51^-EDTA	Measurements of the urinary radioactive EDTA following oral administration of the readily absorbed probe	Non-invasive	Handling of radio-isotope
Confocal laser endomicroscopy (CLE)	Involves injection of a fluorescein contrast agent to the patient and examining the cells and intracellular substance of the gastrointestinal tract mucosa during real-time endoscopy	Real-time observation of intestinal permeability	Invasive. Not readily available. Need special training
Serum biomarkers	Measure the traverse of lipopolysaccharides across the intestinal barrier or shedding of enterocyte-derived proteins	Non-invasive. Easy to perform	Different marker denotes a different endpoint and significance. Variability of methods for measurements. Lipopolysaccharides are also elevated during sepsis

Direct observation of intestinal barrier function may be achieved by confocal laser endomicroscopy (CLE). This is an endoscopic technique with excellent subcellular resolution and 400-fold magnification. The technique relies on injecting a fluorescein as a contrast agent to the patient and examining the cells and intracellular substance of the gastrointestinal tract mucosa during real-time endoscopy [29]. This has been applied successfully to study duodenal permeability in patients with acute pancreatitis [30], and in patients with inflammatory bowel disease [31]. However, its use is limited by the need for special training to perform the test. CLE is also not widely available currently.

Intestinal barrier function may also be examined by serum or plasma intestinal biomarkers. A number of serum markers can be used. Unfortunately, every marker denotes a different endpoint and significance. For example, intestinal fatty acid binding protein (iFABP), an enterocyte-derived protein, serves as a marker of mature enterocyte damage and ischemia [32]. On the other hand, serum measurement of zonulins, an integral part of the intercellular TJs, detects barrier disassembly and increased permeability [33]. Serum citrulline, an amino acid produced by small intestine enterocytes [34], reflects loss of small intestine mass. Finally, LPS measurement in serum or plasma, is elevated when the intestinal barrier is breached [35], but serum LPS is also increased during systemic inflammation and endotoxemia.

Different sizes of Fluorescein Isothiocyanate (FITC)-labeled dextran (dextran 4 k Da, dextran 70 k Da, or a combination of the two) have been used reproducibly for in vivo studies of intestinal permeability in the laboratory to differentiate between pore, leak and unrestricted pathways. This method has not been adapted for clinical evaluation.

### Intestinal barrier disruption in hematologic diseases

Unlike diseases such as non-alcoholic steatohepatitis, inflammatory bowel disease, and autism that have been attributed to a primary breakdown in the intestinal barrier, the increased intestinal permeability associated with hematologic diseases is mostly secondary to the disease process. A compromised intestinal barrier increases the risk of complications in these patients. In this section, we will examine several hematologic diseases in which intestinal barrier functions are disrupted and discuss the clinical consequences of this pathology.

### Sickle cell disease

In the last few years, the integrity of the intestinal barrier has been aggressively investigated in sickle cell disease (SCD), a genetic disorder of mutation, that results from a mutation of the β-globin gene, and renders the sickle hemoglobin (HbS) to polymerization upon deoxygenation, promoting sickling of erythrocytes carrying the HbS. The end-result of the event is the adherence of the sickle erythrocytes to activated leukocytes immobilized on the endothelium, causing microvascular occlusion, vaso-occlusive crisis (VOC), and tissue ischemia. VOC, when involving the splanchnic vasculature, damages the intestinal epithelium [36], as indicated by the elevated serum iFABP levels in SCD [37, 38], and increased intestinal permeability. The local hypoxemia may also be responsible for the altered intestinal microbial composition in SCD [39]. SCD patients exhibit increased abundance of *Veillonella* that correlated with the frequency of VOC [40], and reduced *Alistipes* and *Pseudobutyrivibrio* [41], that are producers of short-chain fatty acids (SCFAs) needed for intestinal health. Intestinal microbial density is also increased in SCD [38, 42]. Changes in bacterial composition and density exacerbate the intestinal injury induced by VOC.

As a result of a breach in the intestinal barrier, SCD patients may experience a vicious cycle of VOC [43]. The increased intestinal permeability allows for the traverse of bacterial products across the barrier to stimulate and activate cellular components that participate in VOC. Not only do SCD patients have higher leukocyte counts [44] and serum biomarkers of neutrophil activation, such as CD64 [45], CD11b/CD18 [46], and CD62L [37, 45, 46] compared to non-SCD individuals, their monocytes [47] and platelets [48] are also activated. Furthermore, circulating aged neutrophils (CANs), a subset of neutrophils with high surface expression of CXCR4 and low CD62L and participate in the VOC process, are also higher in SCD [37, 45, 49]. CANs are regulated by intestinal microbiota [49] through interaction with PAMPs via TLR 2/4 and MYD88 [50, 51]. The direct contact needed for this interaction support the pathogenetic role of increased intestinal permeability in contributing to VOC in SCD.

### Thalassemias

Thalassemias are a group of inherited disorders of the α-globin gene cluster or the β-globin gene cluster involved in Hb synthesis. As a result, ineffective erythropoiesis with subsequent anemia occurs. The primary pathology inciting intestinal mucosal injury is iron overload due to an increased intestinal iron absorption signaled by ineffective erythropoiesis and/or from repeated blood transfusions. Undesired amount of epithelial cell shedding occurs during iron elimination, triggering mucosal permeability [52]. Metabolism of non-transferrin bound iron produces reactive oxygen species that contributes to the cellular dysfunction and apoptosis of enterocytes [53] and results in damage to the TJ complexes and leakage of LPS and (1 → 3)-β-d-glucan (BG), a major component of the fungal cell wall into the circulation [54]. This triggers a hyperinflammatory reaction with the activation of neutrophils and macrophages [55], which may inflict further damage to the intestinal mucosa. The clinical consequences are increased incidence of septicemia and other severe bacterial infections in these patients [56–58].

### Human immunodeficiency virus (HIV) infection

Patients with HIV infections are at higher risks for the development of multiple comorbidities, including cardiovascular and liver diseases [59]. This is believed to be related to chronic immune activation caused by increased microbial translocation from a leaky intestine [60]. Intestinal pathology in HIV occurs at many different levels. The envelop glycoprotein of HIV-1 may directly affect mucosal epithelial cells by upregulating inflammatory cytokines, inducing mucosal injury [61]. Abnormal enterocyte differentiation and increased enterocyte apoptosis due to the failure of the cells to maintain ionic balance and by increased production of interferon-γ (IFNγ) and TNF results in a reduction in the height of the villus and an increase in crypt depth [62]. This has been supported by the demonstration of a decrease in serum citrulline in these patients [63]. Along with an increase in CD8^+^ T cell infiltration of the intestinal lamina propria, increased cytokine production causes damage to the TJs. With the loss of CD4^+^ T cells, especially T_H_17 cells, and B cell dysfunction, there is a decrease in immune modulators and luminal IgA. This facilitates increased microbial growth, TJ disruption, and translocation of luminal content [62]. During acute infection phase, levels of iFABP [64], BG [65], lipopolysaccharide binding protein (LBP), soluble CD14 (sCD14), and endotoxin core antibodies (EndoCAb) are high, indicative of a loss of intestinal barrier function and an increase in the translocation of microbial products. The level of LPS at this stage remains relatively normal suggesting rapidly neutralization of the ongoing LPS translocation [66]. However, during chronic infection, there is a progressive decline in EndoCAb and an increase in plasma LPS. A breakdown in the intestinal barrier functions, therefore, increases the susceptibility of the HIV patients to severe bacterial infections [67] and predispose the patients to the complications due to chronic inflammation and dysregulated immune responses.

### Malaria

Patients with malaria are often complicated by a disruption in the intestinal barrier functions and are predisposed to concurrent septicemia, especially with non-typhoidal salmonella infections [39]. Parasitized erythrocytes are sequestrated in the splanchnic vasculature and cause local ischemia that induces intestinal dysbiosis, and increased intestinal permeability and translocation of bacterial products [68]. Parasite infected erythrocyte sequestration in the GI tract reduces the absorption of L-glutamine [69] needed for enterocyte development, proliferation and maintenance of TJ function [70]. Parasite infected erythrocytes in the lamina propria may also cause GI bleeding [71]. Dysregulated immune response as a consequence of *Plasmodium* infection recruits mast cells to the intestine [69]. Activation of mast cells releases histamine and mast cell proteases that may also damage intestinal barrier. Mast cell protease 1 cleaves occludin 11 in the TJs, and high levels of histamine reported in malaria patients reduces E-cadherin adhesion to promote intestinal permeability [69].

### Intestinal barrier function disruption in oncologic diseases

In contrast to hematologic diseases in which the increased intestinal permeability is due primarily to the disease process, disruption in the intestinal barrier functions in oncologic patients are usually iatrogenic, related to treatment of the underlying conditions, although it may also contribute to the underlying disease. Not surprisingly, the iatrogenic-induced compromised intestinal barrier renders the patients to increased risks for the same range of complications as those observed in hematologic patients, in addition to some distinct end-results. In this section, we will examine several oncologic diseases in which intestinal barrier is breached and highlight the clinical consequences of disruption to the intestinal barrier functions in these patients.

### Chemotherapy-induced disruption of the intestinal barrier

By far, the commonest cause of intestinal mucosal abnormalities in patients with oncologic diseases is chemotherapy-induced disruption of the intestinal barrier. Table 2 shows some of the chemotherapeutic agents well-recognized for inducing mucosal damage. The susceptibility of patients to chemotherapy-induced intestinal injury is also affected by age. Older patients are more likely to be affected than younger patients [72]. Higher chemotherapy dose and use of multiagent chemotherapy regimens also carries higher risks.

**Table 2:** A list of chemotherapy well-recognized for inducing mucosal injury

Class of chemotherapy	Chemotherapeutic agents
Alkylating agents	Busulfan Chlorambucil Melphalan Cisplatin
Anthracyclines	Doxorubicin Daunorubicin
Antimetabolites	Cytarabine Methotrexate 5-Fluorouracil
Antitumors	Actinomycin-D Bleomycin
Podophyllotoxin derivatives	Etoposide
Texanes	Docetexal Paclitaxel
Vinca Alkaloids	Vinblastine Vindesine

**Fig. 3:**
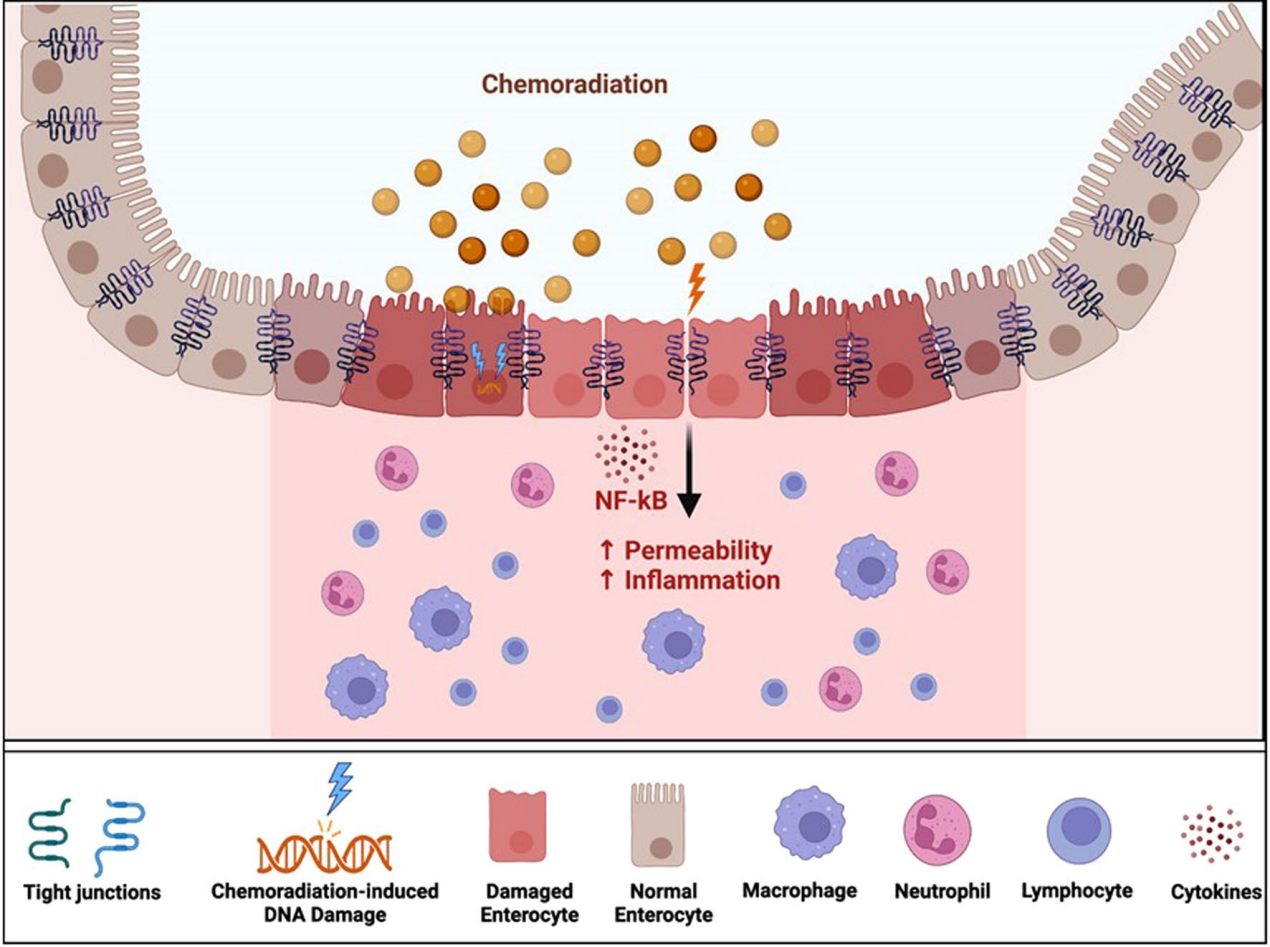
Chemoradiation-induced intestinal mucosal injury in oncologic patients. Chemotherapy affects the intestinal mucosa by its direct and indirect effects on DNA replication and cell growth. In addition, chemotherapy may also directly inhibit the formation of tight junctions

Figure 3 illustrates the cellular and molecular mechanisms associated with chemotherapy-induced intestinal injury. Chemotherapy affects the intestinal mucosa by its direct and indirect effects on DNA replication and cell growth. In addition, chemotherapy may also directly inhibit the formation of TJs. For example, oxaliplatin and irinotecan interfere with TJ formation [73, 74] to increase the intestinal permeability. Both chemotherapeutic agents are used commonly as part of the regimens for treating colon cancer. Major abdominal surgery for colectomy and staging process in these patients further worsens the damage. Methotrexate (MTX), another commonly used chemotherapy, impacts the integrity of TJs in the intestine. A significant decrease in the levels of occludin and claudin-1 proteins occurs 4 days after treatment with MTX [75]. This was not associated with changes in the levels of expression of the corresponding genes, suggesting that MTX affects the localization and cellular expression of TJ proteins, rather than their production [75]. MTX-induced increase in intestinal permeability is mediated by the mitogen-activated protein kinase (MAPK) and nuclear factor kappa B (NF-κB) pathways. The mechanistic effects of chemotherapy on the intestinal barrier functions are, therefore, diverse.

Chemotherapy-induced mucosal injury is also influenced by pro-inflammatory cytokines such as TNF∝, interferon-γ (IFN γ), interleukin 1 (IL-1), and IL-6 [76, 77]. TNF∝ directly affects the function of TJs [77]. It downregulates the expression and impairs the distribution of ZO-1, occludin, claudin-4 and claudin-5, leading to an increase in the barrier permeability over time [78]. IL-1 and IL-6 also induce changes in the TJs [79, 80]. Addition of IL-1 to cell cultures resulted in decreased expression of occludin [81, 82]. Similarly, treatment with IL-6 causes changes in the cytoskeleton, leading to increased permeability across cellular barriers through ZO-1 redistribution, actin structure remodeling, and increased actin contractility [83].

Chemotherapeutic agents may also affect the intestinal barrier functions through inducing alterations in the intestinal microbial community. Patients with chemotherapy-induced intestinal injury have increased abundance of enteropathogenic *E. coli* and *Clostridium perfringens*, both of which are known to disrupt TJ proteins and increase epithelial permeability [84, 85]. Epithelial cells infected with *E. coli* demonstrate increased permeability and destabilization of the TJ proteins ZO-1, occludin, and claudin-1 [84]. Not unlike *E. coli*, *Clostridium perfringens* interact with occludin to result in its redistribution and removal from the TJs [85]. Chemotherapy also decreases the microbial diversity of the intestinal microbiota [86, 87], including producers of SCFAs like butyrate. Butyrate, in particular, is not only needed for enterocyte health and TJ development but also has strong anti-inflammatory effects that reduce intestinal injury and maintain the mucus layer [88–91].

### Infection-associated intestinal injury

Chemotherapy often induces neutropenia, rendering oncologic patients to increased risks for opportunistic infections. The frequent use of broad-spectrum antimicrobials, either for prophylaxis against systemic infections or for the empiric therapy of neutropenic fever, alters the intestinal microbial diversity. As a result, these patients are at increased risks for *Clostridioides difficile* (*C. diff*.) infections and Cytomegalovirus (CMV) colitis, especially in the setting of allogeneic hematopoietic stem cell transplantation. Antimicrobial agents also change intestinal microbiota that may alter the balance in the production of SCFAs vital for the physiologic development of intestinal mucosal functions.

*C. diff.* produces Cytotoxin B that damages the structures within enterocytes by attaching single sugar molecules to proteins that help form actin filaments [92]. As a result of the disarrayed F-actin at the luminal and basal ends of the cell, and the separation of occludin, ZO-1, and ZO-2 from the membrane of lateral TJs, *C. diff*. causes increased intestinal permeability. *C. diff*. infection occurs in 7–14% [93] and recurrent *C. diff*. infections in 11–31% [94, 95] of patients with hematologic malignancies, including acute leukemias, multiple myeloma, Hodgkin’s disease, and non-Hodgkin’s lymphoma.

Immunosuppressed patients are at increased risks for CMV colitis. CMV causes IL-6 mediated damage to the TJs in the intestinal epithelial cells, which leads to a decrease in the transepithelial electrical resistance and an increase in their permeability [96]. As a result, these patients are prone to secondary bacteremia that arises due to increased translocation of bacteria across the intestinal barrier.

Severe neutropenia induced by chemotherapy also predisposes patients to the development of typhilitis or neutropenic enterocolitis in which there is a progressive ascending inflammation of the cecum. Although the pathogenesis of typhilitis is yet to be fully dissected, it is associated with inflammation and mucosal injury, with an increase in intestinal permeability. Consequently, patients are at risk of overwhelming enteric-originated septicemia. Typhilitis is associated with a very high mortality rate [97].

### Cytokine-induced intestinal injury

Since various pro-inflammatory cytokines affect the integrity of the intestinal mucosa, oncologic conditions associated high levels of these cytokines are expected to be associated with a breach in the intestinal barrier functions. Chimeric Antigen Receptor (CAR T)-cell therapy currently approved by the FDA for treating B-cell acute lymphoblastic leukemia [98], B-cell lymphomas [99], and multiple myeloma [100] and is increasingly being administered to patients is associated with cytokine release syndrome (CRS). Similarly, the increasing use of post-transplant high dose cyclophosphamide as a maneuver to reduce the risks for graft-versus-host disease (GVHD) [101] in patients undergoing allogeneic hematopoietic stem cell transplant, especially in peripheral blood stem cell and haploidentical transplants, is associated with high levels of cytokine release during the first 72 h following infusion of the hematopoietic stem cells. Although there have not been any studies investigating the intestinal barrier functions in these two clinical scenarios, it is expected that these patients will likely develop increased intestinal permeability and its associated complications.

### Impaired intestinal barrier functions as driver for underlying oncologic diseases

Although not as extensively studied as in other conditions, a disruption to the intestinal barrier may be a driver for oncologic diseases. In this section, we will discuss how intestinal injury might affect the development of colorectal cancer, progression of myelodysplastic syndrome (MDS), and propagation of acute GVHD following allogeneic hematopoietic stem cell transplantation.

### Intestinal permeability and colorectal cancer

Increase in intestinal permeability may predispose susceptible individuals to the development of colorectal cancer (CRC). This is mediated primarily via inflammatory responses induced by intestinal microbiota that promote tumor development. The intestinal microbiota exploits tumor surface barrier defects to invade and induce inflammation within the colonic tissue [102]. *Fusobacterium nucleatum*, a Gram-negative anaerobic bacterium that adheres to the intestinal mucosa, has been linked to progression of CRC [103]. Its adhesion molecule, FadA, binds to host E-cadherin to enter cells and triggers the WNT/β-catenin pathway, causing the release of cytokines such as IL-6, IL-8 and TNF-α and boosting NF-κB [103]. Interestingly, probiotics, such as *Lactobacillus* and *Bifidobacterium*, may prevent CRC growth by reducing inflammation and blood vessel growth, and by fortifying the intestinal barrier through the production of SCFAs [104]. The breakdown of the intestinal barrier also results in increased risks for the development of bacteremia/septicemia.

Further work supporting the role of intestinal barrier in the progression of sporadic CRC comes from experiments using susceptible mice. A lack of mucus and disruption in the production and localization of TJ proteins result in the entry of bacteria and their products into the tumor stroma to activate Toll-like receptor-mediated inflammatory responses [105]. This process occurs early in colonic tumorigenesis and contributes to the development of CRC through the production of inflammatory cytokines such as IL-23, IL-17A, IL-6, and IL-22 [105]. The role of defective gut barrier in CRC has also been confirmed in mucin 2-knockout (*MUC2*^−/−^) mice in which the lack of gastrointestinal mucin resulted in spontaneous CRC development [106]. Removal of matriptase, a membrane-anchored serine protease that helps form TJs in the intestinal epithelial barrier, specifically in the intestinal epithelium, also leads to the development of colon adenocarcinoma [107, 108].

### Intestinal permeability in the progression of hematologic malignancies

Disruption of the intestinal barrier function may also be associated with the development of hematological malignancies. Bacterial translocation and elevated IL-6 levels caused by small intestine barrier dysfunction are crucial for the development of the pre-leukemic myeloproliferation (PMP) in mice lacking *Tet2*, a gene frequently mutated in hematologic malignancies. Disrupting the intestinal barrier integrity or exposing *Tet2*^−/−^ mice to a systemic bacterial stimulus (e.g. TLR2 agonist) induces PMP in these mice [109].

In patients and mice with acute myeloid leukemia (AML), the normal intestinal microbiome is severely perturbed [110]. They exhibit an imbalance in the intestinal microbial community that results in a reduction of SCFAs needed for the development and health of enterocytes and the production of TJ proteins. Consequently, AML is associated with a decrease in the expression of TJ proteins (claudin-1 and ZO-1) with a resultant increase in the translocation of LPS into the systemic circulation to promote inflammatory responses that favor the progression of the disease [110].

### Intestinal permeability and the propagation of acute graft-versus-host disease

GVHD is a systemic complication that occurs following allogeneic hematopoietic stem cell transplant. It starts with inflammation and tissue injury induced by the high-dose chemoradiation used as the transplant preparative regimens. The intestinal mucosal injury and associated changes in the intestinal microbiome provide the backdrop for the inflammatory processes. Numerous soluble inflammatory proteins result in diffuse, nonspecific damage to numerous organs and the vascular endothelium. Intestinal barrier breakdown allows the traverse of luminal bacteria and bacterial products into the systemic circulation to activate the TLR signaling pathways to worsen the condition. MLCK210 regulates the intestinal permeability. In GVHD, altered MLCK210 is pivotal for the propagation of GVHD-induced intestinal injury, despite lack of changes in the expression of ZO-1 [111]. Mice lacking MLCK210, although may develop GVHD in a Major-Histocompatibility Complex (MHC) mismatched transplant setting, are protected from prolonged intestinal barrier loss and severe GVHD, as shown by reduced tissue damage, decreased number of CD8 + T cells in the gut, and better overall survival [111].

Altered intestinal microbial diversity associated with allogeneic hematopoietic stem cell transplant and low concentrations of SCFAs and lactase, as well as a high level of lactose contribute to the increased intestinal permeability in these patients [112]. A multicenter study that examined the link between a healthy gut barrier and microbiome, and the occurrence of GVHD and its complications found that a diverse microbiome at the time of hematopoietic cell transplantation was linked to a lower death rate related to GVHD [113]. Patients with mild acute GVHD had better gut barrier function and less gut toxicity, as determined by the ^51^Cr-EDTA absorption test [114].

### Strategies to improve intestinal barrier function

Several strategies are available for fortifying the intestinal barrier to modify the disease course or mitigate the potential complications associated with an increase in the intestinal permeability. These strategies may be applicable to all patients, although the choice of approach depends on the clinical scenario.

### Short-chain fatty acids

Intestinal microbiota produces organic acids such as acetate, propionate, butyrate, and valerate through fermentation of dietary microbiota-accessible carbohydrates (MACs) that are non-digestible in the host colon but used by the microbes as an energy source. In the absence of dietary MACs, mucus-degrading bacterial population increases by feeding on mucin. Thus, disruption of the microbial composition will deplete mucus layer, and affect the production of the SCFAs needed for enterocyte health and metabolism, and TJ development. A deficiency of butyrate results in increased intestinal permeability in inflammatory bowel diseases (IBD) [115]. Butyrate also possesses anti-inflammatory properties that may modulate the inflammatory reactions induced by local invasive bacteria. Oral supplement of SCFAs is currently being investigated for hematologic and oncologic diseases (Table 3).

**Table 3:** Clinical studies registered in Clinicaltrials.gov using diet or dietary supplements/changes for hematologic and oncologic patients

NCT#	Study	Primary outcome measurements	Number of patients (n)
04700527	LCCC2032: The effects of short chain tatty acid supplementation on the quality of life and treatment-related toxicities in subjects receiving abdominopelvic radiotherapy: A randomized controlled study	The rate and severity of patient reported and physician determined toxicities between subjects who receive therapeutic SCFA and those who receive placebo	122
05113485	A fiber-diverse, anti-inflammatory diet and aerobic exercise reduce risk of breast cancer recurrence	High-sensitivity C-Reactive Protein (CRP)	30
04538482	Determining the structural-and functional-level effects of diet-specific interventions on the gut-microbiota of a diverse sample of southern United States adults	1) Mean change in alpha diversity of intestinal microbiome; 2). Changes in bile acids; 3) Changes in inflammatory marker IL-6	112
05195970	Microbiota, metabolites and colon neoplasia	1) Bacterial composition and taxonomy changes in the fecal microbiome; 2) Bacterial diversity changes and strain-level variations in the fecal microbiome; 3) Bacterial gene expression profile changes in the fecal microbiome; 4) Urolithin levels in urine; 5) Association of urolithin levels with presence (and type) of colonic lesions; 6) Correlation of urolithin levels with fecal microbiome composition; 7) Correlation of colonic lesion gene expression with urolithin production	200
02446431	Metronomic therapy for pediatric patients with solid tumors at high risk of recurrence: A multi-institutional study	1) 5 year event free survival; 2) Number of participants with adverse events as a measure of safety and tolerability	20
05039060	A modified microbiota-accessible carbohydrates (MAC) diet and change of gut microbiota in patients with colorectal cancer after surgery: A prospective, open-label, cross-over, single center study	The change of gut microbiota diversity	40
05708326	Does timing matter? A case crossover study of intermittent fasting in patients with CLL/SLL at BC Cancer- Victoria	1) Change in lymphocyte count; 2) Change in inflammation; 3) Change in metabolomic profiles; 4) Change in autophagy status; 5) Changes in immune cell gene expression profiles	8

Data from registry as of March 5, 2023

### Pre- and probiotics

Pre- and probiotics supplements are potential approaches to increase intestinal barrier functions [116, 117]. They exert their activities through increasing the expression of TJ proteins, reducing intestinal inflammation, and modulating the local immune responses [118]. They modulate intestinal tissue dendritic cells to produce IL-10, decrease IFN-γ release by intestinal T-cells and decrease LPS-induced production of the proinflammatory IL-12 [119]. Despite a lack of US FDA regulation, the most well-studied products with regards to safety are *Lactobacillus*, *Bifidobacterium*, and *Saccharomyces boulardii*. A major shortfall in these products is that there exists extensive variability in the preparations available, including single or multi-strain products. The other concern on the use of probiotics in immunocompromised patients is the risks for fungemia and bacteremia. A retrospective literature review identified 59 cases of fungemia linked to the use of probiotics [120]. However, a study involving 31 pediatric and adolescent patients undergoing allogeneic hematopoietic stem cell transplant found that administration of *Lactobacillus* from Day -7 during the conditioning regimen through Day + 114 did not result in *Lactobacillus* bacteremia [121]. Dietary changes are being investigated in various hematologic and oncologic diseases (Table 3).

### Fecal microbiota transplant

Fecal microbiota transplant (FMT) has been used successfully for the treatment of recurrent/refractory *C. diff*. infection [122]. It is also being investigated in various diseases associated with a breakdown in the intestinal barrier functions. In addition to restoring the balance of microbial community in the intestine, it provides a mean to replenish the microbial community with SCFA producers to fortify the intestinal barrier. FMT may be therapeutically beneficial to patients with various hematologic and oncologic diseases [123]. In addition to treating malignancy-associated recurrent/refractory *C. diff*. infections, FMT has been used to treat corticosteroid-refractory acute intestinal GVHD and reversal of resistance to immune checkpoint inhibitors in patients with metastatic melanoma. FMT is currently being investigated in various oncologic clinical studies. Table 4 shows a list of studies currently actively recruiting patients with hematologic and oncologic diseases for FMT.

**Table 4:** Clinical studies registered in Clinicaltrials.gov using fecal microbiota transplant in hematologic and oncologic patients

NCT#	Study	Primary outcome measurements	Number of patients (n)
05273255	An open label feasibility study of fecal microbiota transplantation (FMT) in patients with malignancies not responding to Immune Checkpoint Inhibitor (ICI) therapy	Change in the intestinal microbiome community	30
04951583	Phase II trial of fecal microbial transplantation in patients with advanced non-small cell lung cancer and melanoma treated with Immune Checkpoint Inhibitors	Objective response rate in the NSCLC cohort	70
04577729	Inducing remission in melanoma patients with checkpoint inhibitor therapy using fecal microbiota transplantation	Progression free survival	60
05251389	Conversion of unresponsiveness to immunotherapy by fecal microbiota transplantation in patients with metastatic melanoma: A randomized Phase Ib/IIa trial	Efficacy, defined as clinical benefit (stable disease, partial response, complete response)	24
04711967	Prospective study of fecal microbiota transplantation for acute intestinal GVHD after allogeneic hematopoietic stem cell transplantation	1) Change in times of stool; 2) Change in volume of stool	20
04269850	Pilot study of fecal microbiota transplantation in combination with ruxolitinib and steroids for severe acute intestinal graft-versus-host-disease after allogeneic hematopoietic stem cell transplantation	Overall survival	20
04935684	Faecal microbiota transplantation for prevention of graft-versus-host disease after allogeneic stem cell transplantation for haematological malignancies	Graft-versus-host disease and Relapse-Free Survival rate	150
04521075	A Phase Ib trial to evaluate the safety and efficacy of fecal microbial transplantation (FMT) in combination with nivolumab in subjects with metastatic or inoperable melanoma, microsatellite instability-high (MSI-H) or mismatch-repair deficient (dMMR) cancer, or non-small cell lung cancer (NSCLC)	1) Incidence of FMT-related Adverse Events; 2) Overall Response Rate	42
05286294	MITRIC: Microbiota transplant to cancer patients who have failed immunotherapy using faeces from clinical responders	1) Safety evaluation of fecal microbiota transplant; 2) Tumor response evaluation	20
05279677	Phase II, single-arm study of FMT combined with immune checkpoint inhibitor and TKI in the treatment of colorectal cancer patients with advanced stage	Overall response rate	30
04116775	A Phase II single arm study of fecal microbiota transplant (FMT) in men with metastatic castration resistant prostate cancer whose cancer has not responded to enzalutamide + pembrolizumab	Anticancer effect of fecal microbiota transplant from responders to pembrolizumab to non-responders	32
04163289	Preventing immune-related adverse events in renal cell carcinoma patients treated with combination immunotherapy using fecal microbiota transplantation	Occurrence of immune-related colitis associated with ipilimumab/nivolumab treatment	20
04758507	Targeting gut microbiota to improve efficacy of immune checkpoint inhibitors in patients with advanced renal cell carcinoma	Number of participants who will be free from tumor progression	50
04264975	Utilization of microbiome as biomarkers and therapeutics in immuno-oncology	Overall response rate	60
03819296	Role of microbiome in the realm of immune-checkpoint inhibitor induced GI complications in cancer population	1) Difference in stool microbiome pattern; 2) Incidence of adverse events of fecal microbiota transplantation	800
04729322	Pilot trial of fecal microbiota transplantation and re-introduction of anti-PD-1 therapy in dMMR colorectal adenocarcinoma anti-PD-1 non-responders	Objective response rate	15
03819803	Fecal microbiota transplantation in patients with acute gastrointestinal graft-versus-host-disease after allogeneic stem cell transplantation	Gastrointestinal GVHD remission	15
04745221	Efficacy and safety of autologous fecal bacteria transplantation in preventing acute graft versus host disease after haploidentical hematopoietic stem cell transplantation: A multicenter, open, randomized controlled clinical study	Acute graft-versus-host disease	100
04988841	Prospective randomized clinical trial assessing the tolerance and clinical benefit of fecal transplantation in patients with melanoma treated with CTLA-4 and PD1 inhibitors	Safety of a 23 week treatment with MaaT013, combined with ipilimumab + nivolumab in patients with melanoma naïve to Ipilimumab and anti-PD1	60
04975217	Pilot study using fecal microbial transplants in patients with pancreatic cancer	Incidence of adverse events	10
04924374	Microbiota transplant in advanced lung cancer treated with immunotherapy	Measures of safety	20
04769895	Evaluation of the efficacy of MaaT013 as salvage therapy in acute GVHD patients with gastrointestinal involvement, refractory to ruxolitinib; a Multi-center Open-label Phase III trial	Overall response rate of gastrointestinal acute GVHD	75

Data from registry as of March 5, 2023

However, just like probiotics, the risk of introducing infections remains the biggest concern, especially in this group of patients who are usually immunocompromised due to treatment of their underlying malignancies. Fatal extended-spectrum beta-lactamases (ESBL) *E. coli* septicemia was reported in a patient with MDS who received pre-emptive FMT [124].

### Akkermansia muciniphila

*Akkermansia muciniphila (A. muciniphila)* is a Gram-negative obligative anaerobic bacteria that has been linked to various health benefits, including improved gut barrier function and reduced inflammation. Oral supplement of *A. muciniphila* has demonstrated efficacy in patients with metabolic syndrome [125]. However, an overgrowth or over-abundance of the organism may potentially be detrimental in oncologic patients with adenocarcinomas. *A. muciniphila* degrades mucin for its growth. Its abundance may, therefore, induce damage to the mucosal barrier, and promote intestinal permeability. Abundance of *A. muciniphila* may promote metastasis of adenocarcinoma, as has been demonstrated in a hormone receptor-positive breast cancer mouse model [126]. Excessive degradation of the intestinal mucin by the organism may also increase epithelial access by intestinal bacteria to trigger colitis, as has been shown in *Citrobacter rodentium*-induced colitis in gnotobiotic mice [127].

### L-glutamine

L-glutamine is an amino acid considered a “conditionally essential” nutrient. It has been shown to play a role in regulating the intestinal barrier function. L-glutamine not only provides the source of energy for the intestinal epithelium, it also helps maintain the structural integrity of the gut epithelial cells and TJ development [70]. Supplementation with L-glutamine has been shown to improve gut barrier function in animal models and in some human studies, particularly in individuals with gut-related conditions such as IBD and short bowel syndrome [128]. L-glutamine is currently approved by the FDA for treating SCD. However, the biologic rationale for its use in SCD is not based on improving intestinal barrier function but rather to reduce erythrocyte redox imbalance, which triggers sickling of the erythrocytes. Despite this, SCD patients treated with L-glutamine have not shown significant improvement in the parameters for hemolysis [129]. The laboratory findings of breached intestinal barrier functions in patients with SCD [129] raise the possibility that the beneficial effects of L-glutamine in SCD occur via reduction in the intestinal permeability associated with the disease.

L-glutamine has been used in clinical trials to determine its role in reducing chemoradiation-induced mucositis [130, 131]. However, the results have been mixed and may reflect the different dosages and timing of administration of the L-glutamine.

### Keratinocyte growth factors

Keratinocyte growth factors (KGFs) are a group of cytokines that promote the growth and differentiation of epithelial cells, including the intestinal epithelium. Studies have shown that KGFs play a role in regulating the intestinal barrier function by promoting the proliferation and differentiation of gut epithelial cells, and by maintaining the structural integrity of the TJs that form the gut barrier [132]. KGFs have been investigated as a potential therapeutic approach for a variety of diseases and conditions associated with epithelial cell dysfunction. In fact, KGFs have been shown to promote the healing of gut epithelium in individuals with IBD and improve gut barrier function [133]. KGFs have also been shown to improve the survival and proliferation of gut epithelial cells in short bowel syndrome by restoring the intestinal barrier functions [134, 135]. KGFs have been investigated as a potential therapeutic approach for the prevention and treatment of oral mucositis following high dose chemotherapy and autologous hematopoietic stem cell transplant [136]. Patients randomized to the recombinant KGF exhibited lower incidence of severe oral mucositis and reduced incidence of septicemia. KGF is currently licensed for use to prevent chemotherapy-induced mucositis. Unfortunately, its use has been limited by the high benefit: cost ratio.

## Conclusions

There is now abundance of evidence to implicate the roles of increased intestinal permeability in various systemic disease states. In systemic diseases found to be associated with increased intestinal permeability, there remains many questions that needs to be answered before any preventive measures can be formulated. In particular, except for inflammatory bowel disease where there is a definite primary intestinal pathology, it is unclear if the disruption in the intestinal barrier function is primary and be responsible for the systemic diseases or secondary to other causes associated with the primary systemic diseases. If the primary pathology results from a disruption of the intestinal barrier functions, it also remains to be determined what the primary insults are. Various causes have been previously proposed, including nutritional factors, infections and toxins, “hygiene hypothesis”, “lifestyle hypothesis”, and endogenous factors [137]. The application of such factors to explain the pathogenesis of the disease next leads to the question of what then determines the phenotype of the disease that results. Why do some patients develop autism, others Parkinson’s disease, and yet others non-alcoholic steatohepatitis? Unlike the other diseases, the disruption in the intestinal barrier functions associated with many of the hematologic and oncologic diseases are primarily iatrogenic in origin. In these scenarios, the abnormalities may be transient, although may also be unavoidable. Understanding the anatomy and physiology of the intestinal barrier provides the opportunity for therapeutic targeting to either modify the disease course or mitigate the potential complications.

Unfortunately, efforts to accomplish the above aims are hampered by the lack of quality and widely available methods for evaluating and quantitating intestinal barrier functions. Furthermore, while strategies based on sound scientific rationale are available, their efficacy has so far not been translated into successes in the clinic, suggesting the need for further research to address the diagnostic and therapeutic gaps, especially in patients with hematologic and oncologic disease who pose the additional challenge of being immunocompromised.

## Data Availability

Not applicable.

## Abbreviations

51Cr-EDTA: Radioactive chromium complexed with ethylene diamine tetracetic acid
AML: Acute myeloid leukemia
BG: (1 → 3)-β-D-glucan
CANs: Circulating aged neutrophils
CAR T: Chimeric Antigen Receptor
CLE: Confocal laser endomicroscopy
CMV: Cytomegalovirus
CRC: Colorectal cancer
CRS: Cytokine release syndrome
DAMPs: Damage-associated-molecular patterns
ESBL: Extended-spectrum beta-lactamases
EndoCAb: Endotoxin core antibodies
FITC: Fluorescein isothiocyanate
FMT: Fecal microbiota transplant
GI: Gastrointestinal
GVHD: Graft-versus-host disease
HIV: Human immunodeficiency virus
HbS: Sickle hemoglobin
IBD: Inflammatory bowel diseases
iFABP: Intestinal fatty acid binding protein
IFN: Interferon
IL: Interleukin
IgA: Immunoglobulin A
KGFs: Keratinocyte growth factors
L:M: Lactulose:mannitol
LBP: Lipopolysaccharide binding protein
LPS: Lipopolysaccharide
MACs: Microbiota-accessible carbohydrates
MAPK: Mitogen-activated protein kinase
MDS: Myelodysplastic syndrome
MHC: Major-histocompatibility complex
MLCK: Myosin light chain kinase
MTX: Methotrexate
MUC 2: Mucin 2
MYD88: Myeloid differentiation factor 88
NF-κB: Nuclear factor kappa B
PAMPs: Pathogen-associated-molecular patterns
PEG: Polyethylene glycol
PMP: Pre-leukemic myeloproliferation
PRRs: Pattern recognition receptors
SCD: Sickle cell disease
SCFAs: Short-chain fatty acids
TJs: Tight junctions
TLRs: Toll-like receptors
TNF: Tumor necrosis factor
VOC: Vaso-occlusive crisis
